# Early differentiating between the chemotherapy responders and nonresponders: preliminary results with ultrasonic spectrum analysis of the RF time series in preclinical breast cancer models

**DOI:** 10.1186/s40644-019-0248-y

**Published:** 2019-08-28

**Authors:** Fei Li, Yini Huang, Jianwei Wang, Chunyi Lin, Qing Li, Xueyi Zheng, Yun Wang, Longhui Cao, Jianhua Zhou

**Affiliations:** 1 Department of Ultrasound, Sun Yat-Sen University Cancer Center, State Key Laboratory of Oncology in South China, Collaborative Innovation Center for Cancer Medicine, 651 Dongfeng Road East, Guangzhou, 510060 People’s Republic of China; 2 School of Electronic and Information Engineering, South China University of Technology, Guangzhou, 510640 People’s Republic of China; 3 Department of Anesthesiology, Sun Yat-Sen University Cancer Center, State Key Laboratory of Oncology in South China, Collaborative Innovation Center for Cancer Medicine, 651 Dongfeng Road East, Guangzhou, 510060 People’s Republic of China

**Keywords:** Ultrasonic RF time series, Chemotherapy, Treatment response

## Abstract

**Background:**

This study was aimed to assess whether ultrasonic spectrum analysis of radiofrequency (RF) time series using a clinical ultrasound system allows for early differentiating between the chemotherapy responders and nonresponders in human breast cancer xenografts that imitate clinical responding and nonresponding tumors.

**Methods:**

Clinically responding (*n* = 20; MCF-7) and nonresponding (*n* = 20; MBA-MD-231) breast cancer xenografts were established in 40 nude mice. Ten mice from each group received either chemotherapy (adriamycin, 4 mg/kg) or saline as controls. Each tumor was imaged longitudinally with a clinical ultrasound scanner at baseline (day 0) and subsequently on days 2, 4, 6, 8 and 12 following treatment, and the corresponding RF time-series data were collected. Changes in six RF time-series parameters (*slope*, *intercept*, *S1*, *S2*, *S3* and *S4*) were compared with the measurement of the tumor cell density, and their differential performances of the treatment response were analyzed.

**Results:**

Adriamycin significantly inhibited tumor growth and decreased the cancer cell density in responders (*P* < 0.001) but not in nonresponders (*P* > 0.05). Fold changes of s*lope* were significantly increased in responders two days after adriamycin treatment (*P* = 0.002), but not in nonresponders (*P* > 0.05). Early changes in *slope* on day 2 could differentiate the treatment response in 100% of both responders (95% CI, 62.9–100.0%) and nonresponders (95% CI, 88.4–100%).

**Conclusions:**

Ultrasonic RF time series allowed for the monitoring of the tumor response to chemotherapy and could further serve as biomarkers for early differentiating between the treatment responders and nonresponders.

**Electronic supplementary material:**

The online version of this article (10.1186/s40644-019-0248-y) contains supplementary material, which is available to authorized users.

## Background

Among the various treatment modalities for patients diagnosed with malignant tumors, chemotherapy has been reported to be one of the most effective approaches. However, inherent or acquired chemoresistance (i.e., resistance to anticancer drugs) has become a major challenge in cancer therapies that could lead to poor treatment outcomes and survival rates [1]. Therefore, an urgent need exists to explore and verify identifiable imaging biomarkers to early differentiate between drug-sensitive tumors and drug-resistant tumors to decrease unnecessary treatments, avoid adverse side effects and reduce wasted medical resources.

According to the Response Evaluation Criteria in Solid Tumor (RECIST), the evaluation of the treatment response frequently depends on changes in the tumor size measured by computed tomography (CT) or magnetic resonance imaging (MRI) examination after the end of a therapeutic protocol [2]. However, these changes tend to be apparent several weeks to months later, thus limiting the use of the early differentiation between reatment responders and nonresponders. Previous studies have suggested that chemotherapy induce cell death, blood flow decrease and cell metabolism changes, and these changes usually occur before tumor size changes [3]. Consequently, based on the detection of such changes, functional imaging techniques such as dynamic contrast-enhanced CT and MRI, diffusion-weighted MRI and positron emission tomography (PET) have been shown to be promising in the early differentiation of a treatment response in previous studies [4, 5]. Nevertheless, the application of such imaging techniques can be limited by the relative expensive cost, risks of exposure to radiation, possible allergies to contrast agents, and injection of radioactive tracer isotopes.

Ultrasound (US) is relatively inexpensive and lacks radiation risks, which allows for repeatable examination during the treatment course. US imaging systems are also portable and with high resolution on superficial tissues, a special advantage for animal studies and superficial tumors such as breast cancers. Unlike B-mode ultrasound (BUS), which conveys anatomical information, quantitative ultrasound (QUS) conveys tissue microstructure characteristics by quantitatively analyzing the radiofrequency (RF) data backscattered from tissues [6]. Spectrum analysis of single-frame RF signals and RF time-series are the most common types of quantitative ultrasound used for the analysis of ultrasonic RF backscattered signals. Ultrasonic spectrum analysis of single-frame RF data in tissue characterization has been well investigated to date and has been shown to be promising in diagnosing prostate cancer [7], ocular tumors [8], and cardiac abnormalities [9], as well as evaluating the early response to anticancer therapies such as radiotherapy and chemotherapy based on characterizing tumor microstructure changes and cell death in tumor xenografts and clinical patients [10–12].

Unlike spectrum analysis of single-frame ultrasonic RF data, RF time-series analysis is based on the spectrum analysis of sequential RF echo samples acquired from a fixed spatial location in tissue over a short period of time. Daoud et al. suggested that when the ultrasonic wave sequence interacts with the tissue microstructure, the tissue temperature, as well as the sound speed, would change. Thus, RF time-series backscatter signals related to these changes would carry tissue microstructure information [13]. Previous studies have found that the features originated from RF time-series data were significantly more sensitive and accurate than single-frame RF data in tissue characterization [14]. Analysis of RF time-series data has been tested in the detection of liver fibrosis, prostate cancer and breast cancer, as well as in the evaluation of tissue changes after ablation and chemotherapy, and the results were promising [13–21]. Our previous study demonstrated that RF time-series analysis could be used to monitor the microstructure changes after chemotherapy [21]. However, only responding tumor model was used in previous study and it could not fully simulate the clinical situation in which some cancers would be responding to treatment and some would be resistant to treatment. As a results, it is not known whether early changes in the quantitative parameters calculated from RF time series enable the early differentiation between the chemotherapy responding and nonresponding tumors.

Therefore, this study was aimed to assess whether ultrasonic spectrum analysis of RF time series using a clinical ultrasound transducer and system allowed for the early differentiating between the chemotherapy responders and nonresponders in two human breast cancer models, which imitate clinically responding and nonresponding tumors.

## Methods

### Cell culture and animal models

This experiment was approved by the animal care and use committee of Sun Yat-sen University under the guidelines of the National Institutes of Health for the Care of Laboratory Animals. All data supporting the results reported in this study have been uploaded onto the Research Data Deposit public platform (www.researchdata.org.cn) for further reference, with approval number as RDDB2018000283. The human breast cancer cell line MCF-7, which was reported to be adriamycin-sensitive in previous studies [22–24], was used to imitate clinically responding tumors. MBA-MD-231, which was reported to be adriamycin-resistant in previous studies [23–25], was used to imitate clinically nonresponding tumors. Both cell lines were obtained from the State Key Laboratory of Oncology in Southern China and were grown in DMEM medium (HyClone Co., UT, USA) supplemented with 10% fetal bovine serum (Gibco, Grand Island, NY, USA), 50 U/ml penicillin, and 50 μg/ml streptomycin at 37 °C in a humidified 5% CO2 atmosphere. Tumor cells were digested by trypsin and resuspended at approximately 5 × 10^7^ in a 1:1 phosphate-buffered saline and Matrigel mixture (BD Biosciences, San Jose, CA). Tumor cells were inoculated subcutaneously on the right lower hint limb of 5- to 6-week-old BALB/c nude female mice (obtained from Beijing Vital River Laboratory Animal Technology Co., Ltd.) to avoid the interference of heart beating during ultrasound data acquisition.

### Adriamycin treatment

**Fig. 1:**
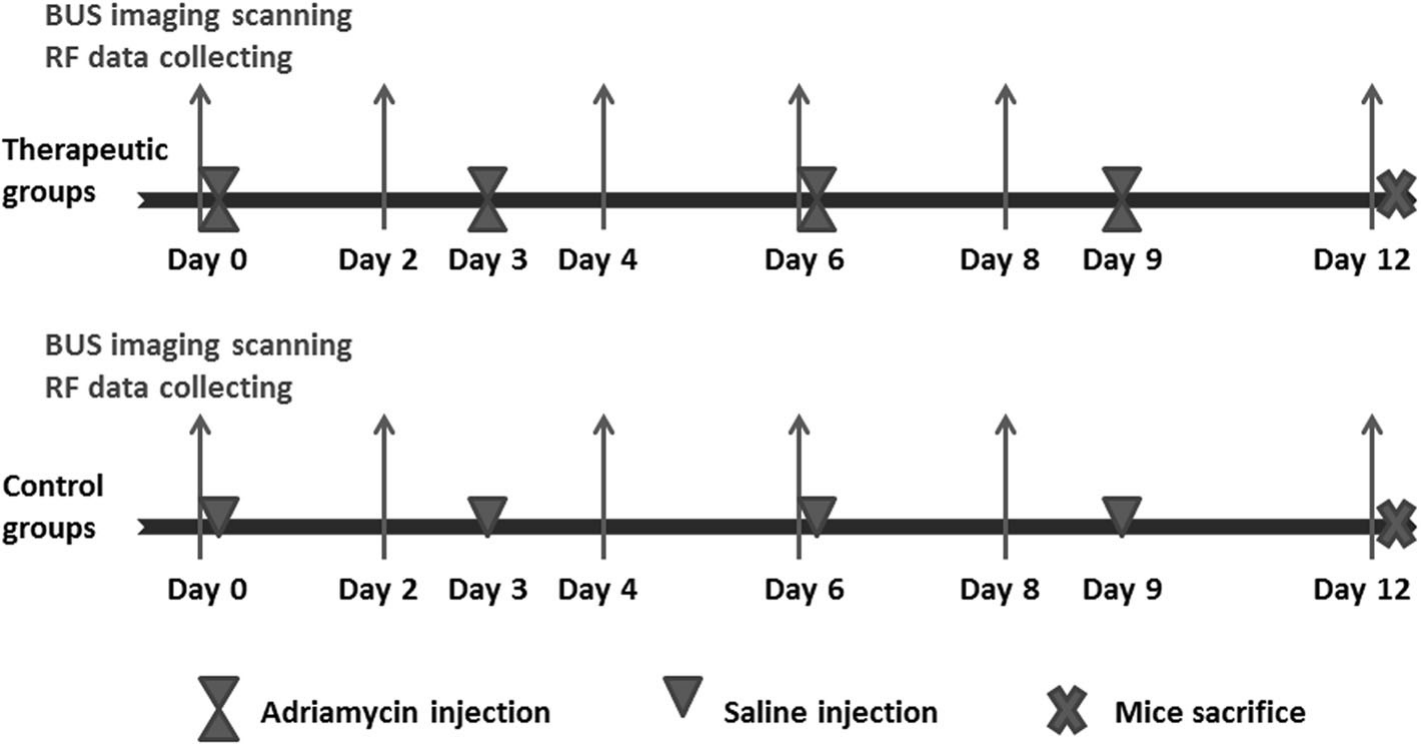
Experiment workflow for both responding (MCF-7) and nonresponding tumors (MBA-MD-231). B mode ultrasound (BUS) scanning and corresponding RF data collecting was performed on days 0, 2, 4, 6, 8 and 12 prior to treatment. Adriamycin or saline was administrated on days 0, 3, 6 and 9. On day 12, all mice were sacrificed and tumors were removed for histopathological examinations

The workflow of the experiment is illustrated in Fig. 1. Nude mice bearing MCF-7 tumors (*n* = 20) and MBA-MD-231 tumors (*n* = 20) were randomly divided into 1) the therapeutic groups (i.e., MCF-7 tumors treated with adriamycin (*n* = 10) and MBA-MD-231 tumors treated with adriamycin (n = 10); 20 tumors total) and 2) the control groups (i.e., MCF-7 tumors treated with sterile saline (*n* = 10) and MBA-MD-231 tumors treated with sterile saline (*n* = 10); 20 tumors total). Mice in the therapeutic group were treated with adriamycin (Shenzhen Main Luck Pharmaceuticals Inc., Guangdong, China) by intraperitoneal injection once every 3 days at the dose of 4 mg/kg, while mice in the control group were treated with sterile saline only. Treatments began when the maximum diameter of the tumor reached approximately 8 mm.

### Ultrasonic data acquisition protocol

All the ultrasound examinations involved in this study were performed by one radiologist who was blinded to treatment conditions. The BUS and RF time-series data were simultaneously acquired on days 0, 2, 4, 8 and 12 using an Acuson S2000 (Siemens, Mountain View, CA) ultrasound system with a linear array transducer at a center frequency of 10 MHz. All animals were anesthetized with 2% isoflurane mixed with room air at the flow of 2 L/min during the whole experiment. Centrifugation of the gel was used to minimize bubble formation in the gel, and a stand-off gel pad was placed on the skin for scanning. BUS imaging was performed to measure the greatest longitudinal (height, H), transverse (width, W) and anteroposterior (length, L) dimensions of the tumors using electronic calipers. The tumor volume was calculated using the formula for a prolate ellipsoid: volume = L × W × H × π × 1/6.

For the acquisition of RF time-series data, QUS imaging was performed in the research mode with the following imaging settings: sampling frequency of 14 MHz, mechanical index of 0.6, frame rate of 41 fps, dynamic range of 65 dB, and imaging depth of 2.5 cm. The largest cross-section plane and two adjacent planes of the tumor were chosen as the imaged specimen to collect RF time-series data. The ultrasound transducer was positioned to make the focal zone at the same depth in each imaged specimen to control for any potential attenuation. With these image settings, 150 consecutive frames of RF data were digitally recorded with the ultrasound probe and tumors fixed in a stationary position for nearly 4 s.

### RF time series data analysis

A self-developed MATLAB-based (v. 2009a: MathWorks, Natick, MA, USA) software system was utilized to analyze the ultrasonic RF time series data. For each tumor image, a rectangular region of interest (ROI) was placed nearly at the focal position of the transducer. The RF time-series parameters from three representative ROIs for each tumor sample were averaged for the final analysis.

**Fig. 2:**
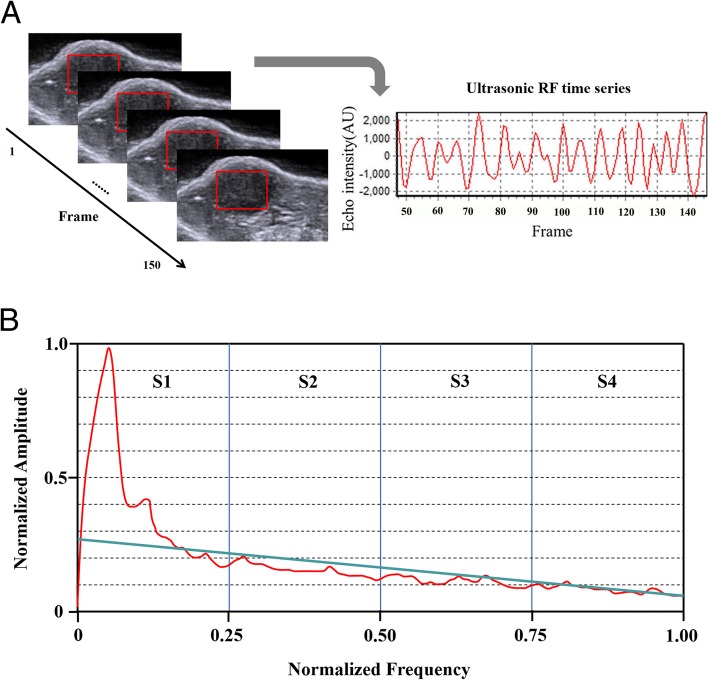
Method of calculating ultrasonic RF time series parameters. **a** An RF time series is formed by temporal ultrasonic signals that are collected continuously from a steadfast ROI (the red rectangle) of tumor tissue. **b** The parameters *S1*, *S2*, *S3* and *S4* were the integral of the normalized average spectrum of the ROI in each quarter of the normalized frequency range (divided by the blue line). The parameters *slope* and *intercept* were the slope and intercept of the regression line (the green line) fitted to values of the spectrum

An RF time series is formed by temporal ultrasonic signals that are collected continuously from a steadfast area of the tumor tissue (Fig. 2a). Based on the method originally reported by Moradi et al. [16], six parameters, s*lope*, *intercept*, *S1*, *S2*, *S3* and *S4*, were calculated to summarize the spectral features of RF time series in this study. The detailed processes of calculating the six parameters have been fully illustrated in the study of Moradi et al. [16]. Succinctly, the six parameters were calculated from the normalized amplitude of RF time series over an ROI using the Discrete Fourier Transformation (DFT). We divided the power spectrum by its maximum to obtain normalized spectral values in the range [0, 1], allowing for comparisons of data from different ROIs. Because all the data were acquired from the same depth, compensation of the depth-dependent attenuations was unnecessary. The parameters *S1*, *S2*, *S3* and *S4* were the integral of the normalized averaged spectrum of the amplitude in each quarter of the normalized frequency range, and the other two parameters were the *slope* and *intercept* of the regression line fitted to values of the spectrum (Fig. 2b). See Additional file 1 for more details.

### Histopathological examination

All mice were sacrificed at the end of the experiment after imaging, and tumors were removed for histopathological examination. Tumor tissues were fixed in 10% buffered formalin before paraffin processing. The tumor specimens were sectioned (5 μm) at the largest cross-sections and stained with hematoxylin and eosin (H&E) to assess changes in the cell density under the microscope. After scanning the H&E stained tumor tissue sections to locate regions with the highest tumor cell density under a × 40-power microscope, ten different fields within the regions of highest tumor cell density were randomly selected under a 400 × −power microscope. The histology images of each 400× field were saved in the computer, and the tumor cell density was measured by calculating the number of nuclei using Image Pro Plus software (image pro-plus 6.0; Media Cybernetics, Silver Spring, MD, USA). The average number of nuclei in ten fields was applied for statistical analysis.

### Data analysis and statistics

We used SPSS version 16.0 (SPSS, Inc., Chicago, IL) to perform all the data analyses. Tumor size and six RF time-series parameters after treatment were normalized to the values of day 0 (before treatment) to show fold changes. The Kolmogorov-Smirnov test was used to analyze normal distribution. The Levene test was used to analyze the homogeneity of variance. Independent Student’s t-test was applied to evaluate differences in the tumor sizes and tumor cell densities that were normally distributed between the therapeutic group and control group in either the responding and nonresponding tumors. Mann-Whitney U test was applied to evaluate differences in the fold changes in six RF time-series parameters that were not normally distributed. The paired-samples Wilcoxon rank test was applied to evaluate differences in the six RF time-series parameters on each day after treatment initiation (days 2, 4, 6, 8 and 12) compared with the baseline (day 0). To evaluate whether an early change in the RF time-series parameters at day 2 compared with day 0 could differentiate chemotherapy responders from nonresponders, the true positive and negative proportions were evaluated, and 95% confidence intervals (95% CI) were constructed using the exact method because of the small sample size. A *P* value < 0.05 was considered significant.

## Results

### Effect of chemotherapy on the tumor volume

All mice were included in the final analysis. No significant differences were found in the mean tumor volume between the therapeutic group and control group for both responding (MCF-7, *P* = 0.449) and nonresponding breast tumors (MBA-MD-231, *P* = 0.733) before treatment (day 0). For MCF-7 breast cancers, the mean tumor volume of the therapeutic group (by 387.96%) was increased significantly less than that of the control group (by 657.26%, *P* < 0.001) at the end of treatment (day 12). The fold changes in the tumor volume of the therapeutic group were significantly smaller than those of the control group on days 6, 8, and 12 (for day 6, *P* = 0.003, for day 8, *P* < 0.001, and for day 12, *P* < 0.001), whereas no significant differences were found on days 2 and 4 (for day 2, *P =* 0.207; for day 4, *P* = 0.165).

**Fig. 3:**
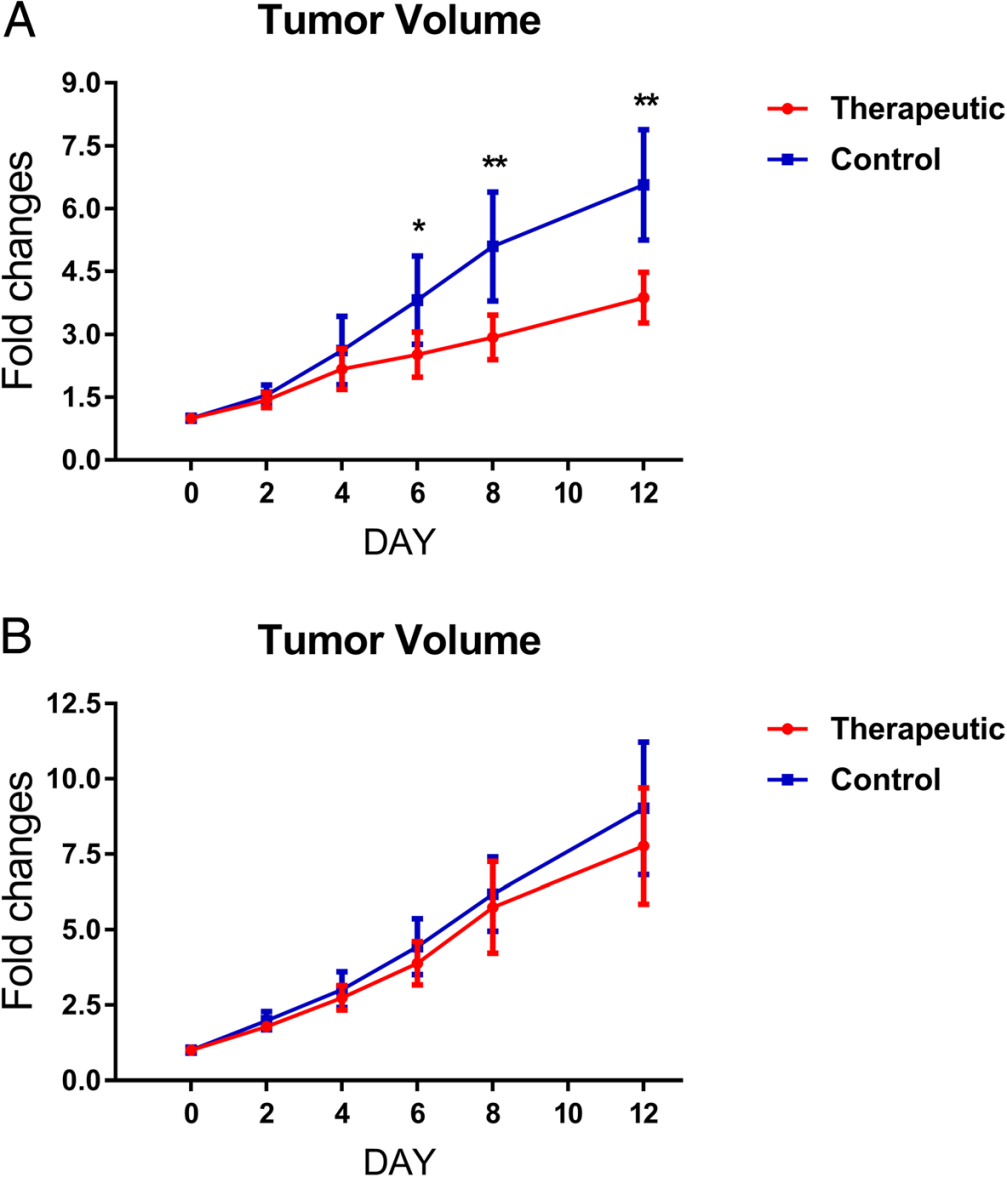
Changes in the tumor volumes in responding and nonresponding tumors. The parameters were shown as the means ± SD. **a** In responders, fold changes in the tumor volumes of the therapeutic group were significantly smaller than those of the control group on days 6, 8, and 12 after treatment initiation (*P < 0.01, ** *P* < 0.001), whereas no significant differences were found on days 0, 2 and 4 (*P* > 0.05). **b** In nonresponders, no significant differences were found regarding the fold changes in the tumor volumes between the therapeutic and control groups on any day (*P* > 0.05)

By contrast, for the nonresponders of MBA-MD-231 breast cancers, the mean tumor volume of both the therapeutic group (by 777.14%) and control group (by 902.95%) were significantly increased (*P* = 0.191) at the end of treatment (day 12). No significant differences were observed in the fold changes of the tumor volume between the therapeutic and control group at the 5 time points post-treatment (*P* from 0.089 to 0.482) (Fig. 3).

### Changes in the RF time-series parameters during the treatment course

**Fig. 4:**
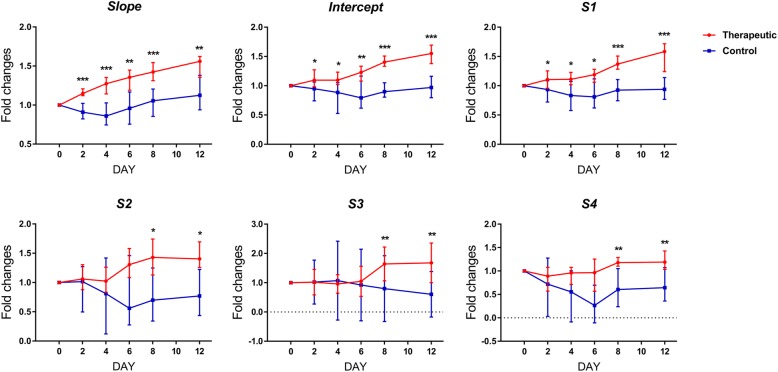
Longitudinal changes in the RF time series parameters in the responding tumors. Bar graph summarize the fold changes of six RF time series parameters compared with baseline. *P < 0.05, ** *P* < 0.01, *** *P* < 0.001 for comparison between control and treated tumors. The parameters were shown as medians and 25th~75th percentiles

**Fig. 5:**
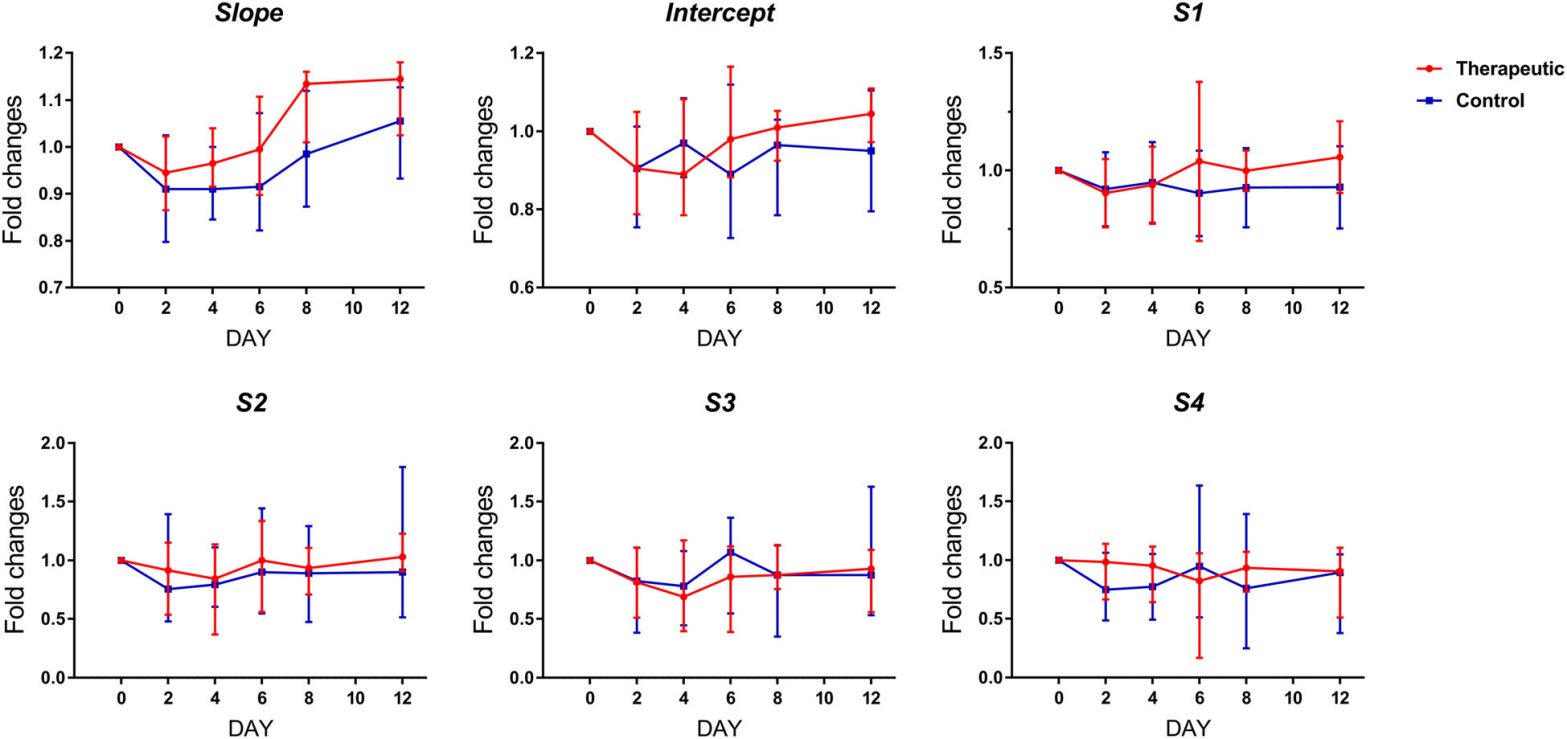
Longitudinal changes in the RF time series parameters in the nonresponding tumors. Bar graph summarize the fold changes of six RF time series parameters compared with baseline. No significant difference was observed in *slope*, *intercept* and *S1*~*S4* on any time points between control and treated tumors (*P* > 0.05). The parameters were shown as medians and 25th~75th percentiles

The fold changes of the RF time-series parameters are shown in Figs. 4 and 5. Before treatment (day 0), no significant differences were found in the *slope*, *intercept* and *S1*, *S2*, *S3*, and *S4* between the therapeutic and control groups in both the responding and nonresponding tumors (*P* from 0.247 to 1.000). In the responding breast cancers of MCF-7, comparing the fold changes in six RF time-series parameters between the therapeutic and control groups, the *slope*, *intercept* and *S1* were significantly increased on days 2, 4, 6, 8 and 12 (for *slope*, *P* from < 0.001 to 0.002; for *intercept*, *P* from < 0.001 to 0.029; for *S1*, *P* from 0.001 to 0.043), while *S2*, *S3* and *S4* were significantly increased on days 8 and 12 (for *S2*, *P* = 0.019 and 0.015; for *S3*, *P* = 0.009 and 0.003; for *S4*, *P* = 0.009 and 0.004) but not on days 2, 4 and 6 (for *S2*, *P* from 0.165 to 0.579; for *S3*, *P* from 0.190 to 0.971; for *S4*, *P* from 0.075 to 0.529). In contrast, in the nonresponding breast cancers of MBA-MD-231, no significant differences were found in the fold changes of the *slope*, *intercept*, *S1*, *S2*, *S3*, and *S4* between the therapeutic and control groups at any time point after treatment (for *slope*, *P* from 0.075 to 0.436; for *intercept*, *P* from 0.353 to 0.912; for *S1*, *P* from 0.190 to 0.971; for *S2*, *P* from 0.796 to 1.000; for *S3*, *P* from 0.436 to 0.912; for *S4*, *P* from 0.353 to 0.739).

Compared with the baseline (day 0), the fold changes of *slope* were significantly increased two days after initiation of chemotherapy and on subsequently time points (*P* = 0.002), and the fold changes of *intercept* and *S1* significantly were increased on days 4, 6, 8 and 12 (*P* from 0.002 to 0.020) but not on day 2 (*P* = 0.084), while the fold changes of *S2*, *S3* and *S4* were significantly increased on days 8 and 12 (*P* from 0.002 to 0.020) but not on days 2, 4 and 6 (*P* from 0.193 to 1.000) in adriamycin-treated responding tumors. By contrast, in saline-treated responding tumors, adriamycin-treated nonresponding tumors and saline-treated nonresponding tumors, no significant difference was found in the fold changes of the six RF time-series parameters at any time point after treatment initiation compared with the baseline (*P* from 0.065 to 1.000).

### Early differentiation between the chemotherapy responders and nonresponders

Because the changes in the RF time-series parameter *slope* occurred much earlier than those in the tumor volume in adriamycin-treated responding tumors, whether the relative change in *slope* at day 2 compared with day 0 could differentiate the chemotherapy responders from nonresponders was assessed. Overall, when using 9.00% or more increase in *slope* two days after adriamycin treatment to differentiate chemotherapy responders from nonresponders, the tumor response to chemotherapy could be early differentiated in 100% of both responders (95% CI, 62.9–100.0%) and nonresponders (95% CI, 88.4–100%).

### Histopathological evaluation of the chemotherapy effect

**Fig. 6:**
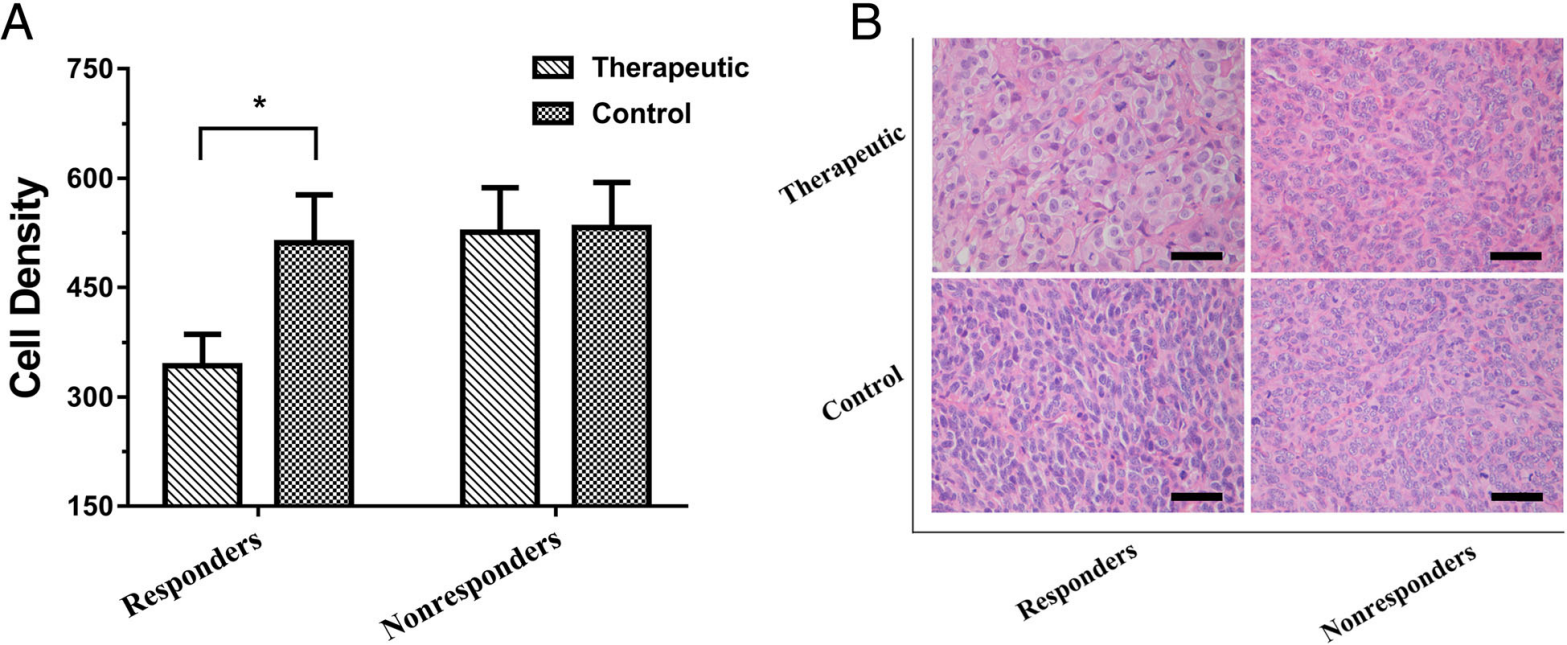
Ex vivo evaluation of the microstructure changes after chemotherapy. **a** Adriamycin treatment significantly decreased the tumor cell density in the responders but did not in the nonresponders. The parameters were shown as the means ± SD (* *P* < 0.001, for comparison between control and treated tumors). **b** Representative H&E-stained tumor micrographs (original magnification, × 400) show tumor cell density which significantly decreased in the treatment responders as compared with the nonresponders. Scale bar, 50 μm

In responders, tumor cell density was significantly decreased in adriamycin-treated tumors compared with saline-treated tumors at the end of treatment (day 12, *P* < 0.001). However, in the nonresponders, no significant difference was found in the tumor cell density between adriamycin-treated and saline-treated tumors at the end of treatment (*P* = 0.818) (Fig. 6).

## Discussion

In the current study, the value of RF time-series analysis in the differentiation between chemotherapy responders and nonresponders was tested in human breast cancer models treated with adriamycin. Our study showed that during the process of a 12-day adriamycin treatment protocol, differences in the tumor volumes could be detected on day 6, while changes in *slope* were evident as early as 2 days after the initiation of therapy. Moreover, an early change in *slope* on day 2 differentiated 100% of treatment responders and nonresponders.

Although chemotherapy is beneficial for most cancer patients, resistance to chemotherapy can lead to poor treatment outcomes. To simulate clinical chemotherapy responders and nonresponders in this study, the human breast cancer cell lines MCF-7 and MBA-MD-231, which have been formerly reported to be adriamycin-sensitive and adriamycin-resistant [22–25], were respectively used. As expected, the tumor growth of adriamycin-treated MCF-7-bearing mice was significantly inhibited compared with that of saline-treated mice, with mean tumor volumes increasing by 387.96 and 657.26%, respectively (*P* < 0.001) at the end of treatment (day 12). Furthermore, histopathological examination proved that adriamycin treatment significantly decreased the tumor cell density of MCF-7-bearing mice when comparing the therapeutic and control groups. By contrast, adriamycin treatment neither significantly inhibited tumor volume growth nor decreased tumor cell density in MBA-MD-231-bearing adriamycin- and saline-treated mice. Our results corroborated the feasibility of using these two different tumor types to represent treatment responders and nonresponders.

Tumor microstructure changes related to cancer cell death, which occurred at the inchoate stage of treatment [3, 26], have long been suggested to be valid biomarkers for early differentiating treatment response in most previous studies [3, 6, 10, 11]. Based on detecting the decrease in cancer cell density and diffusivity of water caused by cancer cell death, diffusion-weighted imaging (DWI)-MRI and its quantitative parameter, the apparent diffusion coefficient (ADC), were utilized for early differentiation and monitoring of the therapeutic response for breast cancer and ovarian cancer patients [6, 27, 28]. However, the relative high cost of MRI examination and low spatial resolution of DWI-MRI limited its extensive application. By contrast, ultrasound imaging systems are relatively inexpensive, portable and show high resolution on superficial tissues, allowing for repeatable examination during treatment. Conventional ultrasound only conveys anatomical information, and its differential performance of treatment response has been reported to be relatively low [6]. Ultrasonic spectrum analysis of RF data backscattered from tissues, including single-frame RF data and RF time-series data, is a novel tissue microstructure characterization technique. The application of single-frame ultrasonic RF spectral analysis for early assessment and differentiation of the treatment response by characterizing chemotherapy-induced tumor microstructural changes has been well investigated by both preclinical and clinical studies [10–12, 29].

RF time-series parameters are fundamentally different from single-frame RF parameters, which are calculated depending on the spectrum analysis of sequential RF echo samples acquired from a fixed spatial location in tissue within several seconds. RF time-series analysis out-performed single-frame RF analysis in tissue typing [13, 14]. Although the underlying mechanism of the enhanced performance is unclear, the cause might be due to the alterations in the tissue temperature as well as sound speed caused by the interaction between the ultrasonic wave sequence and tissue microstructure in the process of the scanning [13]. Such alterations will not occur during single-frame RF scanning procedures because of the instantaneous interaction time. Additionally, another superiority of the RF time series over single-frame RF analysis is that RF time-series analysis needs no signal compensation for depth-dependent effects [30].

Our previous study investigated the usefulness of RF time-series data in the oncology therapy setting, showing that the parameters calculated from ultrasonic RF time-series was significantly changed after chemotherapy and RF time-series parameters were significantly correlated with tumor cell density, which indicated that ultrasonic RF time series analysis could be utilized to detect chemotherapy-induced microstructure changes [21]. However, only treatment responding tumor model was used in previous study to test the possible relationship between changes in RF time-series parameters and underlined microstructure changes, and this could not fully simulate the clinical situation in which some cancers would be responding to treatment and some would be resistant to treatment. In the current study, two different breast cancer mice models were used to simulate clinical responders and nonresponders, respectively, and the ability of RF time series analysis in early differentiation between the chemotherapy responders and nonresponders was tested. The results were promising: an early change in slope on day 2 could differentiate 100% of treatment responders and nonresponders, which implied the possibility of further clinical translation.

We declare some limitations of our study. First, the two-dimensional ultrasound imaging used in this study could not represent the whole tumor volume, which may lead to sampling errors. Hence, we chose the largest cross-section plane and two adjacent planes of the tumor imaging to extract RF time-series data. This would help to reduce bias as much as possible. Further studies should use three-dimensional ultrasound imaging to extract the RF time-series signals of the whole tumor volume, thus improving the detection capability. Second, the amplifier noise effects have not been compensated in the current study. However, a previous analysis showed that with the ROI placement close to the transducer (no more than 3 cm), there was no significant drop in the accuracy of tissue typing [14]. Because the ROIs of our study were placed nearly at the fixed focal position of the transducer less than 2.5 cm, it is reasonable to believe that no compensation was needed for the signal attenuations.

## Conclusion

In conclusion, this preclinical study suggests that ultrasonic spectrum analysis of the RF time series using a clinical ultrasound transducer and system allowed for the early differentiation between the chemotherapy responders and nonresponders in two human breast cancer models that imitate clinical responding and nonresponding tumors. Because ultrasound imaging provided several major benefits such as the relatively low cost, portability and repeatability, and lack of radiation risks, our study built the foundation of further translational research to assess the clinical application of the RF time series to differentiate between the treatment responders and nonresponders in cancer patients without using any contrast agents.

## Additional file

Additional file 1: Detailed process of RF time series data analysis. (DOCX 32 kb)

## Data Availability

All data supporting the results reported in this study have been uploaded onto the Research Data Deposit public platform (www.researchdata.org.cn) for further reference, with approval number as RDDB2018000283.

## Abbreviations

95% CI: 95% confidence intervals
ADC: Apparent diffusion coefficient
BUS: B-mode ultrasound
CT: Computed tomography
DFT: Discrete Fourier Transformation
DWI: Diffusion-weighted imaging
FFT: Fast Fourier Transform
H&E: Hematoxylin and eosin
MRI: Magnetic resonance imaging
PET: Positron emission tomography
QUS: Quantitative ultrasound
RECIST: Response Evaluation Criteria in Solid Tumor
RF: Radiofrequency
ROI: Region of interest
US: Ultrasound
