# Supplementary material for: Early differentiating between the chemotherapy responders and nonresponders: preliminary results with ultrasonic spectrum analysis of the RF time series in preclinical breast cancer models
Source: Cancer Imaging. 2019 Aug 28;19:61. doi: 10.1186/s40644-019-0248-y (PMC6714306; doi:10.1186/s40644-019-0248-y)
Supplement: Supplementary file 1 — Detailed process of RF time series data analysis. (DOCX 32 kb) [file 40644_2019_248_MOESM1_ESM.docx]

**Detailed process of RF time series data analysis**

A self-developed MATLAB-based (v. 2009a: MathWorks, Natick, MA, USA) software system was utilized to analyze the ultrasonic RF time series data. For each tumor image, a rectangular region of interest (ROI) was placed nearly at the focal position of the transducer. The RF time-series parameters from three representative ROIs for each tumor sample were averaged for the final analysis.

An RF time series is formed by temporal ultrasonic signals that are collected continuously from a steadfast area of the tumor tissue (Figure 2A). Based on the method originally reported by Moradi et al., six parameters, s*lope*, *intercept*, *S1*, *S2*, *S3* and *S4*, were calculated to summarize the spectral features of RF time series in this study. The six parameters were calculated from the normalized amplitude of RF time series over an M×N sized ROI using the Discrete Fourier Transformation (DFT). We divided the power spectrum by its maximum to obtain normalized spectral values in the range [0, 1], allowing for comparisons of data from different ROIs. Given that all the data were acquired from the same depth near the focal position, it was unnecessary to compensate the depth-dependent attenuations in this study. The normalized amplitude using DFT can be described as:

$f_{i}^{\text{nor}}=\frac{f'_{i}}{\frac{1}{N_{\text{t}}}\sum_{j=1}^{N_{t}} \left|f'_{j}\right|},f'_{i}=f_{i}-\frac{1}{N_{t}}\sum_{j=1}^{N_{t}} f_{j},i=1,2,\cdots,N_{t}.$ where $f_{i}$ means the amplitude of RF time series in point $i$, and $N_{t}$ means the number of RF time series. The RF time series $f_{i}$ was transformed into $F\left( k \right)$ using Fast Fourier Transform (FFT). Next, the spectrum was averaged over all RF time-series points in one ROI, and the averaged spectrum of the ROI ($F_{\text{ave}}(k)$) was then normalized as follows:

The six abovementioned RF time-series parameters were calculated from $\hat{F}_{\text{ave}}(k)$. The parameters *S1*, *S2*, *S3* and *S4* were the integral of $\hat{F}_{\text{ave}}(k)$ in each quarter of the normalized frequency range:

$\begin{matrix} S_{1}=\sum_{k=1}^{\frac{L}{8}} \left|\hat{F}_{\text{ave}}(k)\right|,S_{2}=\sum_{k=\frac{L}{8}+1}^{\frac{L}{4}} \left|\hat{F}_{\text{ave}}(k)\right| \\ S_{3}=\sum_{k=\frac{L}{4}+1}^{\frac{3}{4}L} \left|\hat{F}_{\text{ave}}(k)\right|,S_{4}=\sum_{k=\frac{3L}{4}+1}^{\frac{L}{2}} \left|\hat{F}_{\text{ave}}(k)\right| \end{matrix}$ where L means the length of RF time series. The other two parameters were the *slope* and *intercept* of the regression line fitted to values of the spectrum (Figure 2B).
